# Comparison of Different NDT Techniques for Evaluation of the Quality of PCBs Produced Using Traditional vs. Additive Manufacturing Technologies

**DOI:** 10.3390/s24061719

**Published:** 2024-03-07

**Authors:** Elena Jasiūnienė, Renaldas Raišutis, Vykintas Samaitis, Audrius Jankauskas

**Affiliations:** 1 Department of Electronics Engineering, Kaunas University of Technology, Studentu St. 50, LT-51368 Kaunas, Lithuania; 2 Prof. K. Barsauskas Ultrasound Research Insititute, Kaunas University of Technology, K. Barsausko St. 59, LT-51423 Kaunas, Lithuania; renaldas.raisutis@ktu.lt (R.R.); vykintas.samaitis@ktu.lt (V.S.); audrius.jankauskas@ktu.lt (A.J.); 3 Department of Electrical Power Systems, Kaunas University of Technology, Studentu St. 50, LT-51368 Kaunas, Lithuania

**Keywords:** PCB, quality, nondestructive testing, additive manufacturing, ultrasonic, radiography, acoustic microscopy, thermography

## Abstract

Multilayer printed circuit boards (PCBs) can be produced not only in the traditional way but also additively. Both traditional and additive manufacturing can lead to invisible defects in the internal structure of the electronic component, eventually leading to the spontaneous failure of the device. No matter what kind of technology is used for the production of PCBs, when they are used in important structures, quality control is important to ensure the reliability of the component. The nondestructive testing (NDT) of the structure of manufactured electronic components can help ensure the quality of devices. Investigations of possible changes in the structure of the product can help identify the causes of defects. Different types of manufacturing technologies can lead to diverse types of possible defects. Therefore, employing several nondestructive inspection techniques could be preferable for the inspection of electronic components. In this article, we present a comparison of various NDT techniques for the evaluation of the quality of PCBs produced using traditional and additive manufacturing technologies. The methodology for investigating the internal structure of PCBs is based on several of the most reliable and widely used technologies, namely, acoustic microscopy, active thermography, and radiography. All of the technologies investigated have their advantages and disadvantages, so if high-reliability products are to be produced, it would be advantageous to carry out tests using multiple technologies in order to detect the various types of defects and determine their parameters.

## 1. Introduction

Multilayer printed circuit boards (PCBs) are an important part of multiple devices [1]. Today, PCBs can be produced not only in the traditional ways, such as layering, photolithography, drilling, and plating, but with recent advances in 3D printing technologies, they can also be produced additively [2,3,4]. Additive manufacturing has the potential to transform electronic component fabrication into a more flexible and customizable manufacturing process, surpassing the limitations of planar designs [5,6,7,8]. Other benefits of the additive manufacturing of electronic components include the efficient use of material resources, waste reduction, and the ability to produce specialized and personalized products on demand. [9]. Potential applications could include the ability to 3D print electronics during space missions [10]. One example of inkjet additive manufacturing technology is the DragonFly LDM electronic sub-assembly technology, which enables the serial production of state-of-the-art electronic PCBs in laboratory conditions by using simultaneous deposition for conductive and dielectric ink [3,5,6]. The additional benefit of designing and manufacturing electronic devices using in-house resources is that, in this way, the risks associated with designing and manufacturing products in external, third-party environments are avoided: this protects against the possible leakage of product technological information, the copying of devices, or the introduction of hidden defects during manufacturing.

The DragonFly LDM equipment uses a newly developed, multi-material deposition technology that has a number of advantages, such as fast device design, safe production, easy product modification, etc. [11]. Nevertheless, there are no specific data on possible structural changes or the degradation of products using the DragonFly LDM manufacturing technology, both during manufacturing and in service. PCBs are produced using multiple composite materials that create the electronic components of the product through the simultaneous deposition of traces using a silver nanoparticle suspension and substrates by using acrylate inks.

PCBs that are part of electronic components in, i.e., military devices, should be reliable and cannot fail unexpectedly [1]. No matter what kind of technology is used for the production of a PCB, when they are used in important structures, their quality has to be ensured.

Traditional and additive manufacturing can cause invisible defects in the internal structure of the electronic component [1], ultimately leading to the spontaneous failure of the device. Quality control for the PCBs is important in order to improve the reliability of these components. Defect detection before the assembly of electronic components can reduce repair costs and help prevent unexpected failures in the future. This is of primary importance when additive processes are used for manufacturing PCBs. Therefore, the nondestructive evaluation of the structure of manufactured electronic components can help ensure the quality of these devices. Investigations into changes in the product’s structure could help identify the causes of defects and modify the additive manufacturing process accordingly until the desired quality has been achieved. However, different types of manufacturing technologies can lead to various types of possible defects; therefore, distinct nondestructive inspection techniques for the inspection of electronic components may be required. PCB quality control is challenging because of the variety of types of defects that can form in components because of mishandling, technical faults in the production process, environmental influence, or human error [1].

There are several NDT techniques that are used for the inspection of PCBs manufactured using traditional technologies. Moreover, it is also known that there is no technique available that would allow for the detection of all types of defects in PCBs [12]. Different techniques are suitable for detecting specific types of defects, each with its own advantages and limitations [12].

Previously, the most popular NDT technique for PCB inspections was manual visual inspection, which is now replaced by automated optical inspection (AOI) [1,13,14]. This technique is based on visual inspection and is good for surface defect detection. Lately, many different image-processing methods have been introduced to make these techniques more efficient [1]. For example, Melnyk et al. implemented thinning and flood-fill algorithms for short and open defect detections in PCBs [15]. In addition, machine learning algorithms and deep learning-based methods such as convolutional neural networks (CNNs) have also been integrated into AOI systems to improve inspection speed and defect detection accuracy [1]. Some studies have presented AI-based inspection to detect corrosion on electronic components [16]. Moreover, in multilayer PCBs, defects can also form at the interfaces between the layers, not only on the surface; so, using AOI, they would remain undetected.

For PCBs manufactured using traditional technologies, radiography is the established inspection technique. Radiographic testing is good for the detection of soldering defects (open or short solder joints, voids in the solder, missing/misplaced components, etc.) [12,17]. Scott et al. presented technology to enhance radiographic inspection by providing material type and thickness information by coupling the analysis of the images with machine learning algorithms [18]. To automatize the evaluation of radiographic images, deep-learning-based methods have been introduced for the detection of solder joint defects [19]. However, digital radiography provides PCB X-ray images with overlapped information [20]: it is challenging to pinpoint from radiographic images in which layer a defect is located. When reconstruction of the images of separate layers of PCBs is required, X-ray-computed tomography is the better option [17]. Nonetheless, image artifacts due to beam hardening and streak artifacts due to metallic components can obscure the images obtained [21].

Acoustic microscopy is especially suitable for the detection of delamination between conductive layers and the substrate [12,22]. Raisutis et al. developed a novel signal-processing method based on the adaptive numerical model to improve the reliability of ultrasound inspection [22]. Wang et al. used laser-induced ultrasound for the detection of delamination-type defects in PCBs [23].

Thermal imaging, specifically, infrared thermography (IRT), has been increasingly used for defect detection in various components, including PCBs [24]. Avdelidis et al. used pulsed thermography for defect detection in PCBs [25], while Wiecek et al. used thermography for the estimation of solder thickness in PCBs [26]. A pulsed thermography-based inspection technique using the digital twin methodology was proposed by Liu et al. for the inspection of electronic components [27].

In addition to the use of single techniques for quality control of PCBs, there have been several attempts to use multiple techniques as well. I.e., Li et al. used a multi-sensor image fusion of polarization information and infrared imaging to improve defect detection accuracy [13]. Nicholson et al. developed a comprehensive online quality control system for PCBs that includes AOI, thermography, digital radiography, and acoustic microscopy [12].

All of these technologies allow us to look inside multi-layered components without disassembling or damaging them and to identify damage to internal structures or deviations from requirements. The inspection of the quality of a PCB before assembly is crucial to understanding whether additive manufacturing technology, in some cases, can be expected to replace traditional PCB manufacturing and provide the same quality of PCBs. Nevertheless, given the relatively small dimensions of electronic components and the extremely small potential damage, this task is not trivial.

No studies were found that compared multiple NDT techniques and their potential to inspect PCBs produced using traditional and additive manufacturing techniques. Such an analysis can provide additional information to the PCB printing technology provider about how their technology works and what quality of products it may produce. Furthermore, it can show how distinct NDT technologies perform when inspecting printed PCBs, which is important for end users. The objective of this work was to compare the potential of different nondestructive testing techniques for the inspection of the quality of PCBs produced using traditional manufacturing technology versus additive manufacturing technology.

In this study, we investigated the potential of multiple NDT techniques, namely, thermography, acoustic microscopy, and radiography, for the inspection of PCBs manufactured using two distinct technologies—traditional and additive manufacturing. For this purpose, a specialized PCB was designed and manufactured using traditional and additive manufacturing technologies. A comparative analysis of different NDT techniques for evaluating the quality of PCBs produced using traditional and additive manufacturing technologies is presented. In addition, defects were intentionally introduced into another additive-manufactured PCB, and a comparison of the ability of different NDT techniques to detect various types of defects is presented. In the following sections, the samples used in this study and the artificial defects are described. Next, the principles of NDT techniques used for the inspection of PCB boards are introduced, followed by a detailed comparison of the inspection results between traditional and additive manufacturing technologies and the defect detection capabilities of additive manufacturing boards.

## 2. Materials and Methods

In order to compare and evaluate the potential of different NDT techniques, a specialized PCB was designed using Altium Designer (San Diego, CA, USA) software package and later produced using traditional and additive manufacturing technology. In addition, in one more additively produced PCB, defects were intentionally introduced. The PCBs produced using different technologies were investigated using the following techniques: thermography, acoustic microscopy, and radiography.

### 2.1. Samples

**Figure 1:**
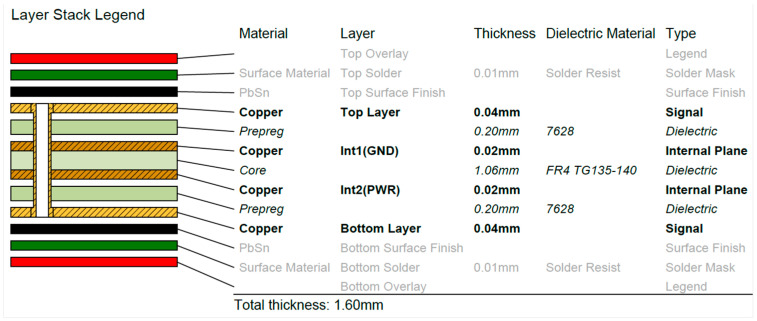
PCB layer stack for the manufacturing of boards using traditional technology.

For a comparison of PCBs manufactured by traditional and additive manufacturing technologies, two sets of boards were prepared. The PCBs were 56 × 65 mm in size and composed of 4 layers with dedicated internal ground and power planes. A layer stack for PCB production using traditional manufacturing technology (layering, photolithography, drilling, and plating) is presented in Figure 1.

Keeping the same stack-up as in the case of manufacturing using the traditional method, the additive manufacturing of PCBs was performed using the DragonFly LDM. The main difference between the manufacturing technologies used was that, for the use of the DragonFly LDM, the base is UV-curable acrylate (Dialectric Ink 1092 NanoDimenion, Waltham, MA, USA). Meanwhile, as a conductive material in PCB production, an AgCite 90072 Silver Nanoparticle Conductive Ink (NanoDimension, Waltham, MA, USA) was used.

**Figure 2:**
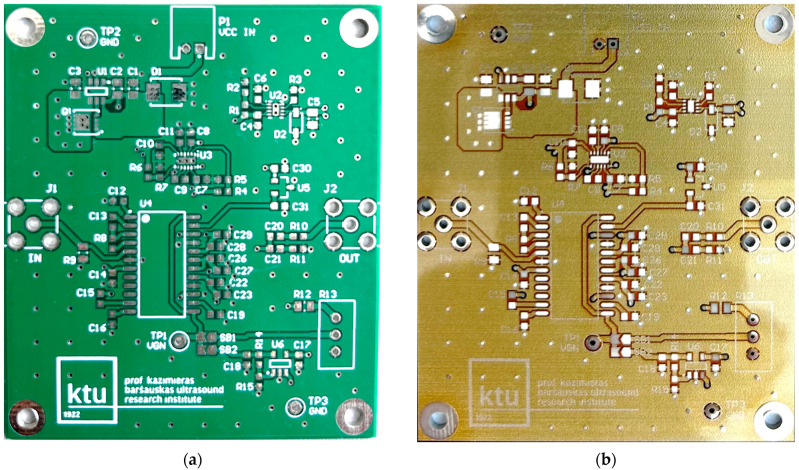
Photos of the PCB boards produced (**a**) using traditional technology and (**b**) with additive manufacturing technology.

**Figure 3:**
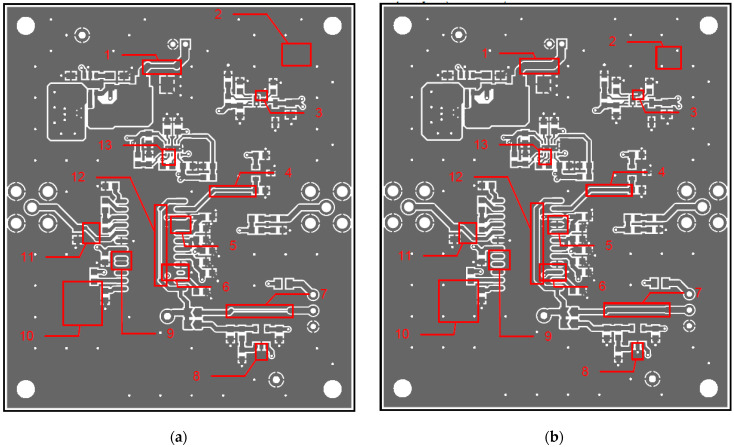
Layout of additively manufactured PCBs (**a**) with defects and (**b**) without defects. Explanation of the numbered zones are given in Table 1.

In Figure 2, photos of PCB boards produced using traditional (a) and additive (b) technologies are presented. In addition to PCBs without defects, artificial defects were introduced into another additively manufactured board. In this case, we chose not to create artificial delamination-type defects, which would imitate disbonding between the conductive layer and substrate. This decision was made due to difficulties in controlling the defect size and position and to ensure the reproducibility of such defects. Therefore, the defects considered were missing vias, tracks, and SMD pads, as well as an incorrect pitch between pads and tracks with width reduction. The positions of the defects are shown in Figure 3, while their description is provided in Table 1.

### 2.2. Thermography

Infrared thermography (IRT) is a non-contact NDT technique that remotely measures temperature variations over the surface of an object of interest, generating a thermal image. This technique relies on detecting the infrared radiation emitted by an object, a process influenced by the object’s temperature, emissivity, surface conditions, and environmental factors [13]. Faults or defects in PCBs can lead to abnormal temperature increases and variations in temperature distribution, which can indicate the presence of potential defects and their respective positions. Two common variants of IRT are available, namely, passive and active thermography, depending on whether an external energy source is used to induce a change in the object’s temperature.

**Figure 4:**
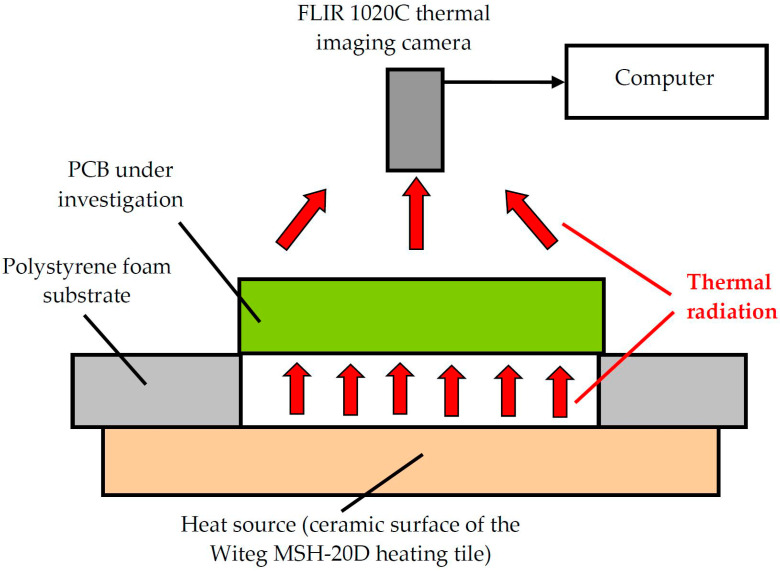
Schematic diagram of the application of a FLIR 1020C high-resolution thermal imaging camera and a Witeg MSH-20D hotplate for printed circuit board (PCB) testing.

In this study, an active IRT technique was used. The printed circuit board (PCB) samples (Figure 2) were examined with a FLIR 1020C thermal imaging camera at high resolution. The number of pixels was 1024 × 768. Thermal sensitivity was <20 mL at +30 °C. The wavelengths of the detected thermal radiation were from 7.5 µm up to 14 µm. The distance between the surface of the test object and the lens of the thermal imaging camera was set to 700 mm. A thermal camera was attached to the photostat to obtain high-quality thermal images and to avoid the effects of blurriness. The sample was placed on a polystyrene foam substrate (12 mm thick), and the substrate was placed on the ceramic surface of a Witeg MSH-20D hotplate (temperature 40 °C). Figure 4 shows a schematic of the experimental setup implementing the active thermography method.

### 2.3. Acoustic Microscopy

**Figure 5:**
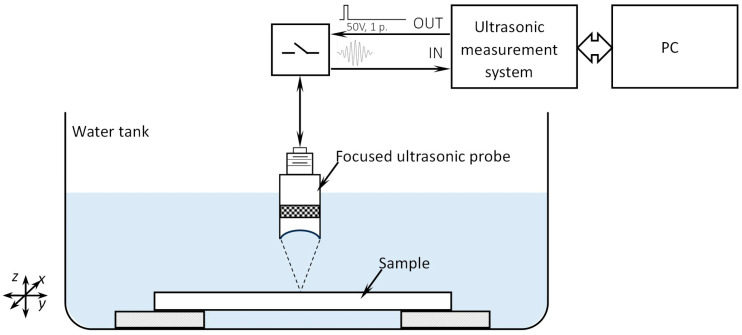
The experimental setup for scanning acoustic microscopy.

Scanning acoustic microscopy (SAM) employs high-frequency ultrasonic waves to examine the internal structure of various components. It allows for the detection and identification of internal delamination, voids, material property changes, and other defects within materials and structures. The working principle of this technique is based on the pulse–echo method (as shown in Figure 5). The probe emits ultrasound waves that penetrate the material. When there are material interfaces, defects, or variations in material density or elasticity, a part of the wave is reflected back to the transducer. Consequently, SAM can create ultrasonic images that display the distribution of reflection magnitudes across the scanning area. The defects will act as unexpected reflectors, whereas changes in material properties will result in varying signal attenuation. SAM is highly sensitive to the presence of delamination, which is difficult to detect using X-ray radiography. It is a recognized technology for nondestructive quality control, inspection, and failure analysis in microelectronic components and materials and is routinely used for the inspection of integrated circuits and other components.

The experimental setup of the system used for the SAM of manufactured PCB boards is presented in Figure 5. For all samples, measurements were conducted in pulse–echo mode on one side of the sample. To ensure acoustic coupling between the sample and the transducer, the measurements were performed in water. The transducer employed for the measurements was a 50 MHz focused Olympus PI200573 transducer with a diameter of 6.35 mm and a focal length in water of 5.08 cm. In all cases, the ultrasonic transducer was focused on the surface of the sample. Ultrasonic signals were collected from the specimen by scanning the surface of the specimen at each scanning point, preserving the transient characteristics of the ultrasonic signals. To enhance the signal-to-noise ratio, the collected signals were averaged 128 times. The ultrasonic transducer was excited with a 50 V pulse, and the received signals were amplified by 21 dB. The received signals were sampled using a 14-bit, 125 MHz analog-to-digital converter. A structural diagram of the measurement is shown in Figure 5. After collecting all the data, C-images of the samples were generated, displaying the maximum of the reflected signal in the scanning plane at each scan position. This image enables the analysis of the signal magnitude distribution on the measured sample, indicating the presence of reflectors at various locations on the printed circuit board.

### 2.4. Radiography

Radiography is an imaging technique that uses X-rays to image the internal structure of the object. During measurements, the X-ray source irradiates the test object with a cone beam, and a 2D image at the detector is recorded. The inspected PCB is placed between the radiation source and a detector. The object absorbs a certain number of X-rays. Absorption depends on the density and thickness of the object. A thicker and/or denser area will stop more of the radiation. X-rays pass the object attenuated, and 2D projectional images at the detector are recorded. X-rays ‘see’ a defect as a thickness variation, and the larger the variation, the easier the defect is to detect. The darkness of the image will vary with the amount of radiation reaching the detector through the test object.

When the path of the X-rays is not parallel to a defect, the thickness variation is smaller, and the defect may not be visible. Since the angle between the radiation beam and a linear defect is critical, the orientation of the defect must be well known if radiography is to be used to perform the inspection.

**Figure 6:**
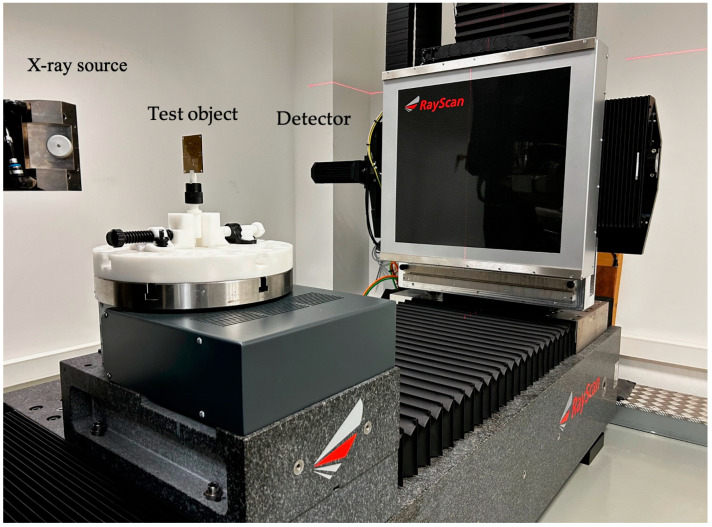
Photo of the experimental setup for radiographic testing.

For radiographic testing, a Rayscan 250E computed tomograph was used. Investigations were carried out with a 225 kV microfocus tube. The image was recorded using a 2048 × 2048 flat matrix detector. A schematic of the investigation setup used is shown in Figure 6. The investigations were carried out using a voltage of 150 kV, a current of 130 µA, and an integration time of equal to 3000 ms. The focal spot using these parameters is 10 μm with a voxel size of 35 μm. However, the resolution achieved is dependent on the size of the investigated object.

## 3. Results and Discussion

### 3.1. Comparison of Multiple NDT Techniques for the Inspection of PCBs Produced Using Traditional and Additive Manufacturing Technologies

**Figure 7:**
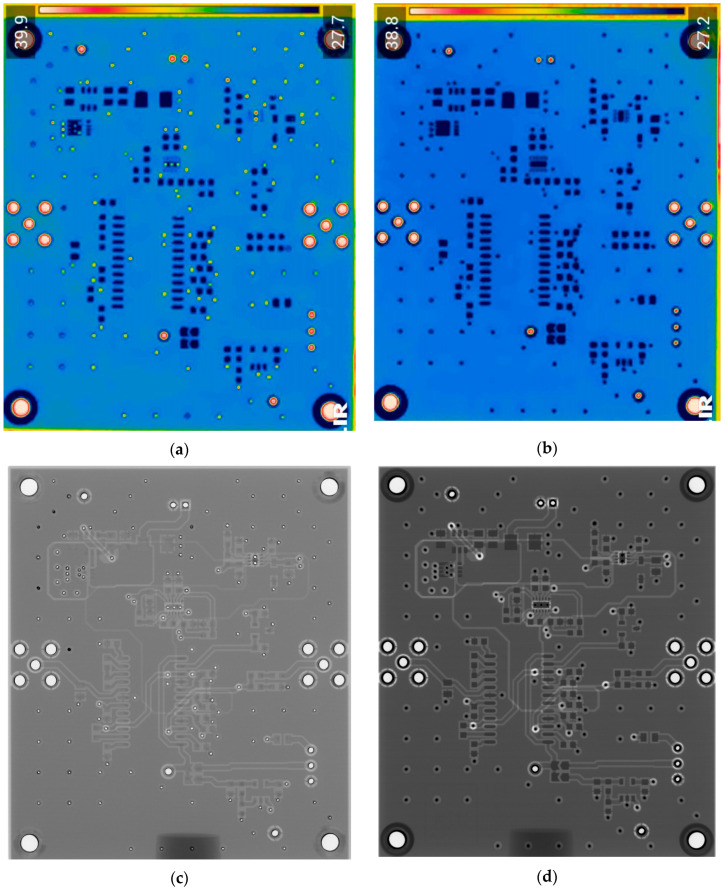

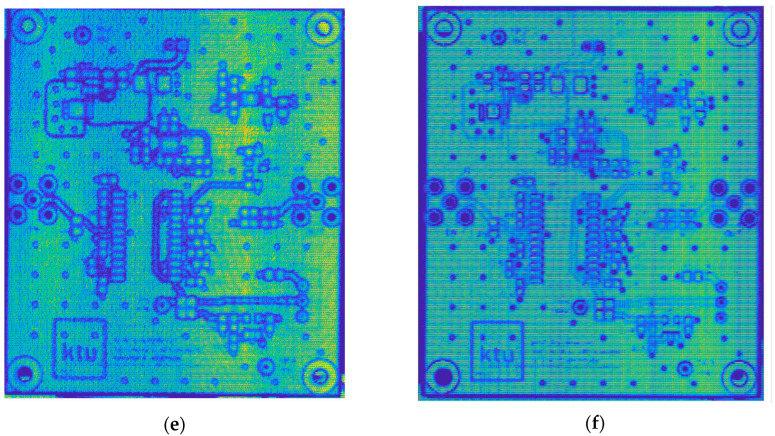
Comparison of images obtained by different NDT techniques of the same PCBs produced using traditional and additive technologies: (**a**) thermal image of the traditionally produced PCB; (**b**) thermal image of the PCB produced using additive manufacturing technology; (**c**) radiographic image of the traditionally produced PCB; (**d**) radiographic image of the PCB produced using additive manufacturing technology; (**e**) ultrasonic image of the traditionally produced PCB; (**f**) ultrasonic image of the PCB produced using additive manufacturing technology.

In Figure 7, images obtained with multiple techniques using the same PCBs produced using traditional and additive technologies are presented. Photos of the PCBs produced using different technologies are presented in Figure 2. Figure 7a is a thermal image of the traditionally produced PCB, and in Figure 7b, a thermal image of the PCB produced using additive manufacturing technology is presented. Radiographic images are presented in Figure 7c of the traditionally produced PCB, and Figure 7d shows the PCB produced using additive manufacturing technology. Ultrasonic images are shown in Figure 7e of the traditionally produced PCB, while Figure 7f shows the PCB produced using additive manufacturing technology.

It could be observed that, in the thermographic image of the additively manufactured board, the vias have the same temperature (dark blue color) as the pads, whereas, in the traditionally manufactured board, the vias have a higher temperature—they have a yellow color. This may indicate that the vias in the 3D-printed PCB have a smaller diameter compared with the traditional PCB. This is suggested by the higher via temperature in the traditional PCB, which implies better heat transmission from the hotplate located beneath the sample. In general, the thermal images were unable to indicate tracks, while the radiographic image showed a higher contrast between the traces and the base material in the additively manufactured board. This indicates that the base material of the 3D-printed PCB has lower emissivity. Thermal cameras have limited spatial resolution in lateral directions and are unable to detect very small defects. This limitation is related to the number of active pixels in the thermal camera’s matrix, as well as the optics and spatial dimensions of the sample being inspected. The results of thermography inspections depend on the thermal conductivity of the materials being inspected. Sufficiently small internal defects may not generate enough heat to reach the surface of the sample and be emitted as thermal radiation. These effects can restrict the detection of defects that are located in deeper layers of the PCB. The materials used in the sample being investigated have varying emission coefficients, resulting in different thermal radiation and making the interpretation of the obtained image more complex. Additionally, the results of thermography inspections are affected by environmental factors such as unstable ambient temperature and random heat flows.

The radiographic images (Figure 7c,d) clearly demonstrate that images of the additively manufactured PCB board have higher contrast when scanned with the same parameters. This higher contrast significantly improves the evaluation of the information contained in the image and could facilitate automatic analysis. It is important to note that the radiographic images obtained have the highest resolutions—for the PCB samples inspected, it was possible to achieve a 35 μm resolution. If one could inspect not the whole board but a part of it, it would be possible to achieve a resolution of up to 10 μm. Radiographic inspection has two main drawbacks: ionizing radiation poses a risk to human health, and inspection equipment is expensive. Nevertheless, it also has some advantages, such as the ability to provide images with the highest resolution and a quick process. With the inspection parameters set up in advance, the investigation of one PCB can be completed in just 3 s.

The ultrasonic images of the traditionally produced and additively manufactured PCBs (Figure 7e,f) showed a similar response. However, it can be seen that. when using the same inspection setup, the tracks are easier to observe in the traditionally manufactured PCB. The reason may lie in the fact that additively manufactured PCBs use very small amounts of conductive material, which produces material property variations that are harder to detect using the selected inspection setup. However, it is important to note that the provided insights are valid for the particular inspection setup, and changes in inspection parameters may lead to varying results. In general, ultrasonic inspection can be considered the slowest technique, as it requires scanning the surface of the sample and usually submerging the sample in water. The inspection resolution highly depends on the inspection frequency. In this case, by using a 50 MHz frequency, we were able to achieve a 150 μm resolution. By increasing the inspection frequency, the resolution can go as high as 20 μm. On the other hand, unlike thermographic inspection, ultrasonic inspection allows for better visualization of the internal structure, while, in contrast to radiographic inspection, ultrasound provides better capabilities in detecting delamination defects.

### 3.2. Comparison of Different NDT Techniques for the Inspection of PCBs Produced Using Additive Manufacturing Technology

**Figure 8:**
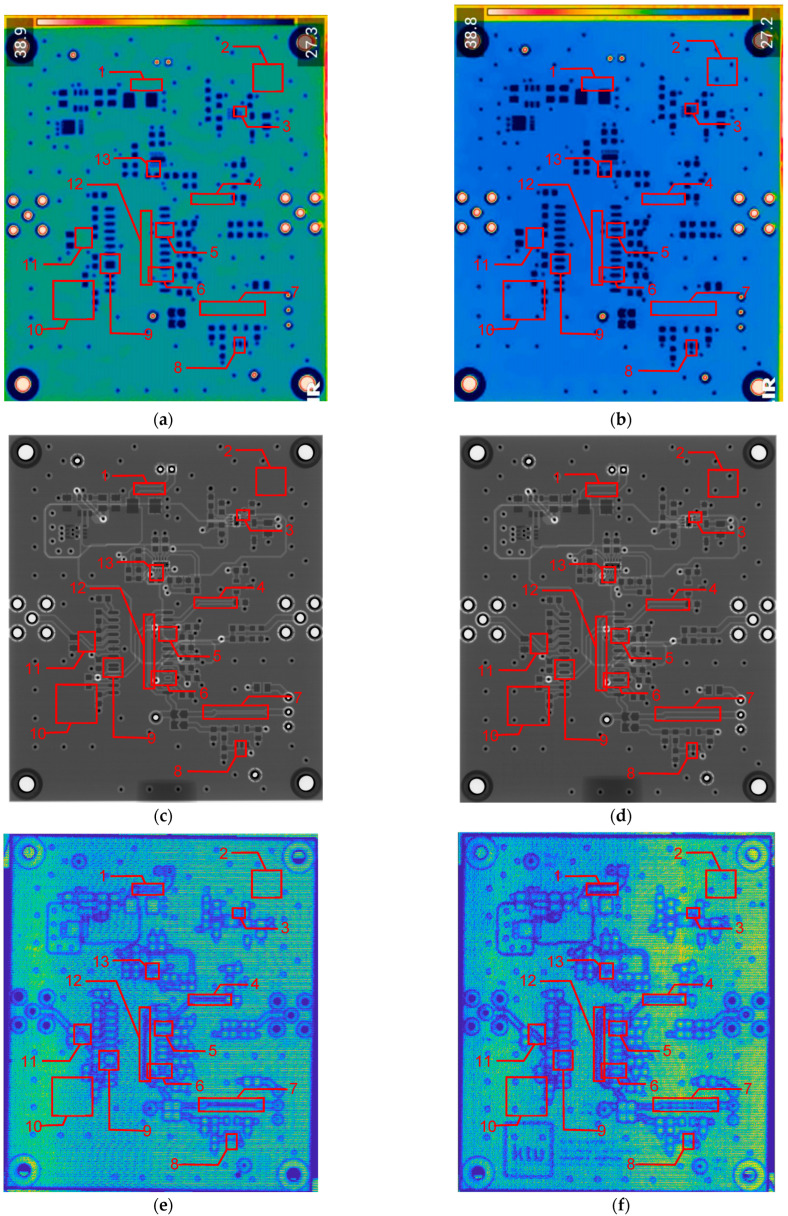
Comparison of images obtained by multiple NDT techniques of the good and defective PCBd produced using additive technology: (**a**) thermal image of the defective PCB; (**b**) thermal image of the good PCB; (**c**) radiographic image of the defective PCB; (**d**) radiographic image of the good PCB produced using additive manufacturing technology; (**e**) ultrasonic image of the defective PCB; (**f**) ultrasonic image of the good PCB produced using additive manufacturing technology. Explanation of the numbered zones are given in Table 1.

In Figure 3, the layouts of the good and defective PCBs produced using additive technologies are presented. In Figure 8, images obtained using distinctive NDT techniques are presented of the good and defective PCBs produced using additive technology. Figure 8a shows a thermal image of the defective PCB, and in Figure 8b, a thermal image of the good PCB is presented. Radiographic images are presented in Figure 8c of the defective PCB and in Figure 8d of the good PCB. Ultrasonic images are shown in Figure 8e of the defective PCB, and Figure 8f shows the good PCB. In Table 2, we summarize which defects are visible and which are not using different techniques. It can be concluded that radiography allows us to find all artificially created defects, and a 35 μm resolution can be achieved. While the ultrasonic inspection allowed us to achieve a 150 μm resolution, defects 3 and 13 were not visible, namely, a missing track between the SMD component pad and the exposed pad of the component (defect 3) and a missing track between the SMD component pads (defect 13); despite this, the width of both of them was 0.2 mm. This result can be explained by the ultrasound attenuation in the inspected PCBs, where especially high-frequency components tend to be filtered, reducing the actual inspection frequency. Thermography resolved the least number of defects, with a spatial resolution of 0.1 mm according to the lateral directions. With thermography, it was possible to detect all the missing vias (defects 2 and 8), tracks (defect 3), SMD components (defect 5), and pad size reduction (defect 6).

## 4. Conclusions

In this study, a comparison of different NDT techniques for the evaluation of the quality of PCBs produced using traditional and additive manufacturing technologies was presented. A methodology for investigating changes in the internal structure of PCBs is based on several of the most reliable and widely used technologies, namely, acoustic microscopy, active thermography, and radiography. The use of several nondestructive technologies ensured the reliability of the screening since defects with diverse natures can be detected in this way. Our study shows the following:

1. Using active thermography, the contact component solder joints on the PCB surface are visible and can be separated from each other. The spatial resolution according to the lateral directions was 0.1 mm. The thermal images obtained show that the contact component solder joints on the surface of the PCB (various shapes: rectangular and square), as well as the vias, are clearly visible and can be distinguished from each other. Our investigations show that thermography can be used to perform the fast detection of defects in the metallization of the surface contacts (damage to contact tracks, squares, and shielding areas,). The limitations of thermal camera applications are related to the limited spatial resolution and sensitivity of the matrix, the thermal conductivity and emissivity of different materials used in the sample being inspected, the depths of defect locations, and the effects of environmental factors on radiated heat.
2. Using SAM, the internal structure of printed circuit boards made using both traditional and Dragonfly LDM technology can be reproduced. The resolution achieved in the sample depends on the thickness of the sample, the smoothness of the surface, the frequency used, and the scanning step. Using a 50 MHz focused transducer and a scanning step of 0.15 mm, contact patches and tracks as small as 0.5 mm can be seen best. In general, the technique was able to detect missing vias, tracks, and SMD pads, as well as incorrect pitch between pads and tracks with width reduction. Under the selected inspection setup, SAM was unable to detect defect 3 or defect 13, which were missing track defects and were too small. However, increasing the inspection frequency and reducing the scanning step would lead to better resolution. On the other hand, such an inspection would be even more time-consuming, as it would require scanning the sample surface with a step size of less than 0.1 mm. In this study, the defects were only considered errors in conductive layer depositions, so SAM was unable to reveal its full potential. It is expected that the presence of delamination between conductive layers and substrates would be perfect for detection with SAM. It should be noted that, in the case of multilayered PCBs, the conductive layers, i.e., the power and ground planes, obscure the reflections from the objects located underneath. In cases where the tracks are located on both sides of the PCB, measurements may need to be taken on both sides of the sample. SAM inspection possesses the longest inspection times and requires the sample to be submerged in water. However, it has a better ability to inspect the internal structure of the sample compared with thermography and has a greater potential for detecting delamination defects compared with radiography.
3. High-resolution X-ray images can be obtained using radiography, and resolutions of 100–10 μm can be achieved depending on the parameters and size of the specimen. However, it should be noted that the resolution achieved may vary with changes in the object to be examined (the number of layers in the plate, the density of the materials the plate is made of, or the thickness of the plate)—the resolution achieved with X-rays also depends on the parameters and size of the object being examined. Radiography cannot distinguish the plane in which the objects are visible, but with reference images, it would be possible to quickly assess deviations caused by the manufacturing. Radiographic images of additively manufactured PCB boards have better contrast when scanned with the same settings. This improved contrast makes it easier to evaluate the information in the images and simplifies the automatic analysis. It is important to mention that radiographic images have the highest resolution form of all the investigated technologies. The advantages of radiographic inspection are the ability to provide the highest-resolution images and the speed of the process. With preset inspection parameters, a PCB can be inspected in as little as 3 s.

All of the technologies investigated here have their advantages and disadvantages, so if high-reliability products are to be produced, it would be advantageous to carry out tests using different technologies in order to detect the various types of defects and to determine their sizes and depths. Each method used in this study results in the visualization of the internal structure of a PCB. These images of the internal structure could be utilized for automated defect detection algorithms, ranging from simple metrics, such as 2D correlation analysis, to more sophisticated object detection algorithms using artificial intelligence. In this way, the quality assessment process for PCBs could be partly automated.

## Figures and Tables

**Table 1:** Artificial defects created in additively manufactured PCBs.

Detail View No.	Defective PCB	Kind of Defect/Imperfection	Defect-Free PCB
1.	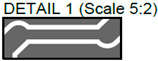	Track width reduction from 1 mm to 0.5 mm.	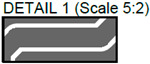
2.	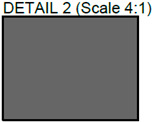	Missing stitching vias (polygon pour (GND)-to-GND plane connection).	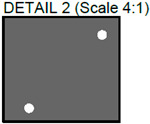
3.	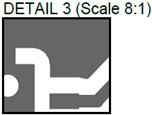	Missing track between the SMD component pad and the exposed pad of the component.	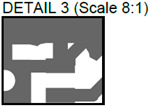
4.	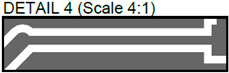	Track width reduction from 0.5 mm to 0.25 mm.	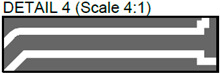
5.	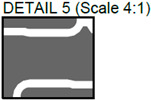	Missing SMD component pad.	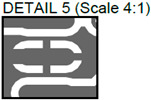
6.	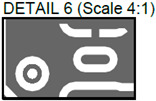	Pad size reduction by 50%; no connection between pad and via.	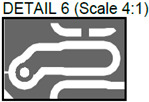
7.	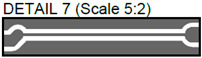	Track width reduction from 0.6 mm to 0.127 mm.	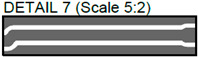
8.	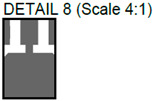	Missing via (polygon pour (GND)-to-GND plane connection).	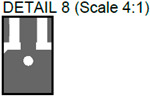
9.	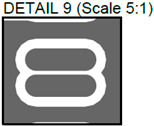	Pitch between pads changes from 1.27 mm to 0.7 mm.	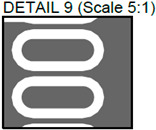
10.	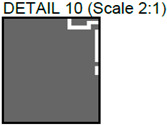	Missing vias (polygon pour (GND)-to-GND plane connection).	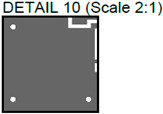
11.	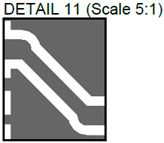	Track width reduction from 0.5 mm to 0.35 mm.	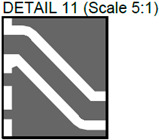
12.	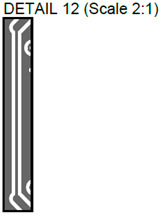	Track width reduction from 0.5 mm to 0.127 mm.	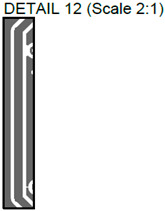
13.	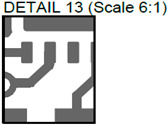	Missing track between SMD component pads.	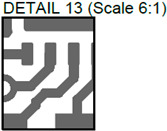

**Table 2:** Detectability of various types of defects using different techniques in additively manufactured PCBs.

Defect Number	Defect Description	Detectable with Thermography	Detectable with Ultrasonic	Detectable with Radiography
1	Track width reduction from 1 mm to 0.5 mm.	N	Y	Y
2	Missing stitching vias (polygon pour (GND)-to-GND plane connection).	Y	Y	Y
3	Missing track between the SMD component pad and the exposed pad of the component.	Y	N	Y
4	Track width reduction from 0.5 mm to 0.25 mm.	N	Y	Y
5	Missing SMD component pad.	Y	Y	Y
6	Pad size reduction by 50%; no connection between pad and via.	Y	Y	Y
7	Track width reduction from 0.6 mm to 0.127 mm.	N	Y	Y
8	Missing via (polygon pour (GND)-to-GND plane connection).	Y	Y	Y
9	Pitch between pads changes from 1.27 mm to 0.7 mm.	Y	Y	Y
10	Missing vias (polygon pour (GND)-to-GND plane connection).	Y	Y	Y
11	Track width reduction from 0.5 mm to 0.35 mm.	N	Y	Y
12	Track width reduction from 0.5 mm to 0.127 mm.	N	Y	Y
13	Missing track between SMD component pads.	N	N	Y

## Data Availability

The data presented in this study are available on request from the corresponding author.
